# A Label-Free Quantitative Proteomic Analysis of Mouse Neutrophil Extracellular Trap Formation Induced by *Streptococcus suis* or Phorbol Myristate Acetate (PMA)

**DOI:** 10.3389/fimmu.2018.02615

**Published:** 2018-11-13

**Authors:** Xiaoping Wang, Jianqing Zhao, Cong Cai, Xiaojuan Tang, Lei Fu, Anding Zhang, Li Han

**Affiliations:** ^1^State Key Laboratory of Agricultural Microbiology, College of Veterinary Medicine, Huazhong Agricultural University, Wuhan, China; ^2^The Cooperative Innovation Center for Sustainable Pig Production, International Joint Research Center for Animal Disease Control, Wuhan, China; ^3^Key Laboratory of Development of Veterinary Diagnostic Products, Ministry of Agriculture, Wuhan, China

**Keywords:** proteomic analysis, neutrophil extracellular traps (NETs), *Streptococcus suis*, phorbol myristate acetate (PMA), MMP-8

## Abstract

*Streptococcus suis* (*S. suis*) ranks among the five most important porcine pathogens worldwide and occasionally threatens human health, particularly in people who come into close contact with pigs or pork products. An *S. suis* infection induces the formation of neutrophil extracellular traps (NETs) *in vitro* and *in vivo*, and the NET structure plays an essential role in *S. suis* clearance. However, the signaling pathway by which *S. suis* induces NET formation remains to be elucidated. In the present study, we used a label-free quantitative proteomic analysis of mouse NET formation induced by *S. suis* or phorbol myristate acetate (PMA), a robust NET inducer. Greater than 50% of the differentially expressed proteins in neutrophils infected by *S. suis* showed similar changes as observed following PMA stimulation, and PKC, NADPH oxidase, and MPO were required for NET formation induced by both stimuli. Because PMA induced robust NET formation while *S. suis* (MOI = 2) induced only weak NET formation, the association between the inducer and NET formation was worth considering. Interestingly, proteins involved in peptidase activity showed significant differential changes in response to each inducer. Of these peptidases, MMP-8 expression was obviously decreased in response to PMA, but it was not significantly changed in response to *S. suis*. A subsequent study further confirmed that MMP-8 activity was inversely correlated with NET formation induced by both stimuli. Therefore, the present study provides potentially important information about the manner by which neutrophils responded to the inducers to form NETs.

## Introduction

Neutrophils are the most abundant white blood cells in the circulation and serve as the first line of immune defense against a pathogen attack (1). Neutrophils kill microbes through phagocytosis, degranulation, the production of reactive oxygen species (ROS), and the secretion of antimicrobial proteins (2). In addition, neutrophils also attack pathogens using a novel antimicrobial mechanism called a neutrophil extracellular trap (NET) (3). This mechanism is initiated following induction with phorbol myristate acetate (PMA) (3), bacteria (4, 5), fungi (6), or viruses (7, 8) when the lobular shape of the neutrophilic nucleus is lost. The nuclear envelope is subsequently disintegrated, resulting in the mixing of the nucleus and the cytoplasmic and granular components of the cell. The cell membrane is then ruptures, resulting in the release of decondensed chromatin into the extracellular space and the subsequent formation of 15- to 25-nm chromatin fibers (3, 4). The fibers interact with high concentrations of histones and antimicrobial molecules (9), providing the NET with the ability to restrict pathogens at the site of infection and to ultimately destroy any pathogen it contacts (10, 11). In addition to the antimicrobial property of NETs, the exposure of self-DNA and the extracellular presence of potentially damaging granule proteins may also have pathological consequences. Indeed, NETs are linked with a variety of inflammatory conditions, including sepsis, small vein vasculitis, and pneumonitis (12).

Mechanistically, NET-mediated cell death (NETosis) is distinct from apoptosis and necrosis (4), and was initially recognized to be dependent on the generation of ROS by NADPH oxidase (4). The activation of conventional protein kinase C (PKC) was essential for the induction of NET formation by PMA, an activator of PKC and a robust NET inducer (13), but it is not essential for NET activation induced by *Helicobacter pylori* (14). The Raf-MEK-ERK pathway mediates the activation of NADPH oxidase (14), and the ROS-dependent activation of ERK and p38 MAPK (15) is involved in NET formation induced by PMA. Calcium flux has been implicated in the generation of ROS and contributes to NET formation induced by PMA (16), but it is dispensable for NET formation induced by *Candida albicans* and Group B *Streptococcus* (17). ROS generation is a hallmark of NETosis induced by PMA (4). However, ROS production does not appear to be required for NET formation induced by Group B *Streptococcus* or nigericin (17). The selective release of neutrophil elastase (NE) and myeloperoxidase (MPO) from the azurophilic granules is required for PMA-induced NET formation (6, 18). In addition, the autophagy process (19), kindlin-3-β2-integrin signaling (20), actin polymerization (21), glycolysis (22), and Rab27a (23) are also reported to be involved in NET formation through a mechanism independent of *de novo* gene expression (24). Based on these observations, the signaling pathway responsible for NETosis is complicated and remaines to be elucidated.

*Streptococcus suis* (*S. suis*) is a major swine pathogen that is responsible for severe economic losses in the porcine industry and representes a significant threat to human health (25–27). The infection in humans leads to meningitis, sepsis, arthritis, endocarditis, and endophthalmitis, and the pooled case-fatality rate is ~12.8% (28).

Since the first reported case of *S. suis*-induced meningitis in humans in Denmark in 1968, more than 1,600 human infection cases have been reported worldwide (28, 29). In addition, *S. suis* has also been recognized as the leading and second leading cause of adult meningitis in Vietnam and Thailand, respectively (25, 30, 31). For a many years, *S. suis* infections in humans remained sporadic and mainly affected individuals with close contact with pigs or pig-derived products (32–34). However, the two large-scale outbreaks in China (35, 36) and human cases without a history of animal contact (37, 38) have modified the opinion regarding the threat of this pathogen to humans. According to an *in vitro* study, *S. suis* induces the formation of NETs (5, 39), and the capsular structure (5), extracellular DNase (39), and biofilms (40) contributed to the evasion of NET-mediated pathogen killing. However, *in vivo* studies further confirmed that *S. suis* induces NET formation (41, 42), which contributes to the clearance of *S. suis* during an infection (41). Thus, NETs play an essential role in the control of *S. suis*-mediated diseases. However, the mechanism by which *S. suis* induces NET formation has not been extensively explored.

A label-free quantitative proteomic analysis is a very powerful tool for studying protein alterations (43), and it has been widely used to analyze the host cellular response to stimulation. In the present study, a label-free quantitative proteomic method was used to analyze the response of neutrophils to an *S. suis* infection. Because PMA is a well-recognized NET inducer (4, 13), the PMA-induced alterations in levels of proteins involved in NET formation were also determined. By comparing the responses of neutrophils to *S. suis* infection and PMA induction, we attempted to provide information about the proteins involved in the NETs formation induced by *S. suis* infection or PMA stimulation, and lay the foundation for further characterization of the mechanism underlying NETs induction.

## Materials and methods

### Bacterial strains and growth conditions

The epidemic *S. suis* strain 05ZY was isolated from the brain of a diseased piglet during the *S. suis* outbreak in China in 2005 (44). The *S. suis* strains were maintained on Tryptic Soy agar (Difco Laboratories, Detroit) plus 10% bovine blood or cultured statically in tryptone soy broth (Difco Laboratories, Detroit) plus 10% bovine blood to mid-log phase (OD at 600 nm of 0.4) at 37°C under aerobic conditions.

### Ethics statement

C57BL/6 mice were purchased from the Laboratory Animal Center of Hubei Province (Permit Number: 42000600007746). The mice were euthanized using CO_2_ to avoid suffering before neutrophil isolation. The study was performed in strict accordance with the Guide for the Care and Use of Laboratory Animals Monitoring Committee of Hubei Province, China, and the protocol was approved by the Committee on the Ethics of Animal Experiments at the College of Veterinary Medicine, Huazhong Agricultural University (Permit Number: HZAUMO-2016-042). All efforts were made to minimize the suffering of the animals used in the study.

### Isolation and purification of mouse bone marrow neutrophils

Mouse bone marrow neutrophils were obtained from 30 specific pathogen-free C57BL/6 mice (10- to 15-week-old) and purified using previously described method (45). Bone marrow from the femurs and tibias was flushed with HBSS-Prep [Ca-Mg-free HBSS supplemented with 20 mM NA-HEPES (pH 7.4) and 0.5% FCS] with a 25-gauge needle. The whole bone marrow aspirate was centrifuged, and the RBCs were hypotonically lysed with 0.2% NaCl. The solution was restored to isotonicity with 1.2% NaCl and then filtered over a 70-μm nylon cell strainer. The solution was centrifuged, and resuspended in HBSS-Prep, and then applied to a 62% Percoll gradient (prepared in HBSS-Prep). The Percoll solution was centrifuged at 1,000 g for 30 min. At the end of the gradient-centrifugation, a sharp interface containing immature cells and non-granulocytic lineages formed atop the 62% Percoll layer, and a cloudier pellet (the neutrophils) was also present. The cells at the interface of the HBSS-Prep and the upper part of the 62% Percoll were carefully removed and discarded. The cell pellet was transferred to another tube, washed twice with 1640 RPMI, resuspended in medium and counted.

The purity of the neutrophils was detected by flow cytometry using the procedure described below. The isolated mouse bone marrow neutrophils were fixed with 4% paraformaldehyde. Prior to staining, the Fc receptors were blocked with a rat anti-mouse CD16/32 antibody (BioLegend, No. 101302). Cells were then stained with an allophycocyanin (APC)-conjugated anti-mouse Ly-6G antibody (BioLegend, No. 108411). Finally, the cells were analyzed using a BD FACSVerse™ flow cytometer and FlowJo 7.6.1 software.

### Induction of NETs formation by SS2 strains or PMA

The isolated neutrophils (10^6^ cells) were seeded into six-well cell culture plates in 2 ml of RPMI 1640 and then incubated with 100 nM PMA (sigma, Cat. No. P8139), *S. suis* (MOI = 2), or PBS. At 60, 120, 180, and 240 min post-inoculation, NET formation was visualized by staining cells with a 100 nM solution of the extracellular nucleic acid dye SYTOX Green (Invitrogen, S7020), observing the staining under a fluorescence microscope (20 × objective, OLYMPUS IX70) and determining the absorbance with a BioTek synergy HT plate reader at excitation/emission wavelengths of 485/530 nm as decribed previously (46, 47).

The isolated neutrophils were incubated with 100 nM PMA, *S. suis* (MOI = 2), or PBS for 4 h. Subsequently, these cells were fixed with 2% (wt/vol) paraformaldehyde (Sigma-Aldrich) and then incubated with the extracellular nucleic acid dye SYTOX Orange for 10 min. After washing, cells were further incubated with a Ms mAb against myeloperoxidase (FITC) (Abcam, ab90812) for 1 h. After five washes, all slides were mounted with 50% glycerol and covered with glass cover slips, followed by an analysis with a LSM 880 confocal microscope (ZEISS) and ZEN 2.3 LITE software (ZEISS). Wavelengths of 488 nm and 561 nm were used because the excitation/emission wavelengths of FITC and SYTOX Orange are 488/520 and 547/570 nm, respectively.

Neutrophils (6 × 10^6^ cells) were plated on a Petri dish in 8 ml of RPMI 1640 and then incubated with 100 nM PMA, *S. suis* (MOI = 2), or PBS. The supernatants were removed, and the cells were lysed with 200 μL SDT buffer (4% SDS and 100 mM Tris-HCl, pH 8.0). The total protein was extracted and subjected to SDS-PAGE analysis and a label-free quantitative proteomic analysis.

### Label-free quantitative proteomic analysis

An aliquot containing 100 μg of extracted proteins was incubated with 100 mM DTT at 100°C for 5 min then mixed with 200 μL of UA buffer (8 M urea and 150 mM Tris-HCl, pH 8.0) in a hyperfiltration tube (Microcon units, 10 KD). After centrifugation at 14,000 g for 15 min, the proteins were alkylated in 100 μL of 50 mM iodoacetamide in UA buffer for 30 min in the dark. The filters were washed twice with 100 μL of UA buffer and then twice with 100 μL of 25 mM NH_4_HCO_3_ buffer. Subsequently, the proteins were incubated with 40 μL of trypsin buffer (2 μg trypsin in 25 mM NH_4_HCO_3_ buffer) at 37°C for 16–18 h. Then, the peptides were collected in a new tube for the LC-MS/MS analysis.

The peptides were desalted on an Empore™ SPE Cartridges C18 (standard density, Sigma), dried by vacuum centrifugation and reconstituted in 40 μL of 0.1% (v/v) formic acid. The peptide mixtures were loaded onto a reverse phase trap column (Thermo Scientific Acclaim PepMap100, 100 μm × 2 cm, nanoViper C18) connected to the C18 reversed-phase analytical column (Thermo Scientific Easy Column, 10 cm long, 75 μm inner diameter, 3 μm resin) in buffer A (0.1% formic acid) and separated with a linear gradient of buffer B (84% acetonitrile and 0.1% formic acid) at a flow rate of 300 nl/min controlled by IntelliFlow technology. The gradient of buffer B was from 0 to 45% for 10 min, followed by a gradient from 45 to 100% for 8 min, and then a hold at 100% for 12 min.

The LC-MS/MS analysis was performed on a Q Exactive mass spectrometer (Thermo Scientific) that was coupled to Easy-nLC (Thermo Fisher Scientific) for 120 min. The mass spectrometer was operated in positive ion mode. MS data were acquired using a data-dependent top 10 method that dynamically choose the most abundant precursor ions from the survey scan (300–1,800 m/z) for HCD fragmentation. The automatic gain control (AGC) target was set to 3e6, and the maximum injection time was set to 10 ms. Dynamic exclusion duration was 40.0 s. Survey scans were acquired at a resolution of 70,000 at m/z 200, the resolution for HCD spectra was set to 17,500 at m/z 200, and the isolation width was 2 m/z. The normalized collision energy was 30 eV and the underfill ratio, which specifies the minimum percentage of the target value likely to be reached at maximum fill time, was defined as 0.1%. The instrument was run with peptide recognition mode enabled.

The data were analyze using MaxQuant software (Max Planck Institute of Biochemistry in Martinsried, Germany) and was based on the UniprotKB mouse database (83,914 total entries, downloaded 07/11/17). The following parameters were considered for the searches: missed cleavages: 2; precursor mass window: 6 ppm; MS/MS tolerance ppm: 20; de-isotopic: TRUE; fixed modification: carbamidomethyl (C), variable modification: oxidation (M) and acetylation (protein N-terminus); decoy database pattern: reverse; label-free quantification (LFQ): TRUE; LFQ min ratio count: 1; iBAQ: TRUE; match between runs: 2 min; peptide FDR ≤ 0.01; and protein FDR ≤ 0.01. The mass tolerance allowed for the precursor ions was 2.0 Da, and for fragment ions was 0.8 Da. For species with high evolutionary homology, shared peptides in razor peptide can affect quantitative results, but shared peptides have little effect on overall database searching and quantification for species that have evolved distantly, such as mouse and *S. suis* in the present study. Razor peptides were selected as a small supplement of median normalization for quantitatively unique peptides to obtain additional data. For comparisons between samples, LFQ was performed with a minimum of a ± 2.0-folder change to determine the differentially expressed peptides.

### Confirmation of the proteomic analysis results by a western blot analysis

A sample containing 80 μg of the extracted proteins from the NETs induced by PMA, *S. suis* or PBS was subjected to a Western blot analysis with antibodies against PGRP-S (sigma, SAB2500783), WDR5 (sigma, PLA0256), Lysozyme (abcam, ab158508), Calpain (abcam, ab108400), or MMP8 (abcam, ab81286). The expression of GAPDH was also determined as a reference control with a GAPDH antibody (Calbio, CB100127).

### Bioinformatics analysis

The differentially expressed (DE) proteins were analyzed with DAVID Bioinformatics Resources 6.7 (http://david.abcc.ncifcrf.gov for a functional classification of these proteins.

### Observation of NET formation in the presence of inhibitors

The toxic effects of inhibitors were initially determined to confirm that the indicated concentrations of these inhibitors would not cause obvious cell death. The isolated neutrophils were seeded in 24-well plates at a density of 10^5^ cells/well and then pre-treated with the inhibitors [1 μM Ro 31-8220 (Sigma, R136), 5 μM DPI (Sigma, D2926), 100 μM ABAH (Sigma, A41909), 10 μM cytochalasin B (Sigma, C2743) or 10 μM MMP8 inhibitor I (Calbiochem, 444237)] for 30 min before stimulation with 100 nM PMA or *S. suis* (MOI = 2). Cells without stimulation served as a control. NET formation was visualized by staining with a 100 nM solution of the extracellular nucleic acid dye SYTOX Green (Invitrogen, S11368) for 10 min and then measured in a BioTek synergy HT plate reader at excitation/emission wavelengths of 485/530 nm. Cells were incubated with SYTOX Orange for 10 min to further investigate NET formation. After washing, cells were further incubated with Ms mAb against myeloperoxidase (FITC) (Abcam, ab90812) for 1 h. After five washes, all slides were mounted with 50% glycerol and covered with glass cover slips, followed by an analysis with an LSM 880 confocal microscope (ZEISS) and ZEN 2.3 LITE software (ZEISS). Wavelengths of 488 nm and 561 nm were used because the excitation/emission wavelengths of FITC and SYTOX Orange are 488/520 and 547/570 nm, respectively.

### Statistical analysis

The data were analyzed with an unpaired, non-parametric, *t*-test, and all of the assays were repeated at least three times. The data were expressed as the mean ± standard error of the mean (SEM), and a value of *p* < 0.05 was considered the significance threshold.

## Results and discussion

### NET formation is induced by PMA stimulation or *S. suis* infection

**Figure 1:**
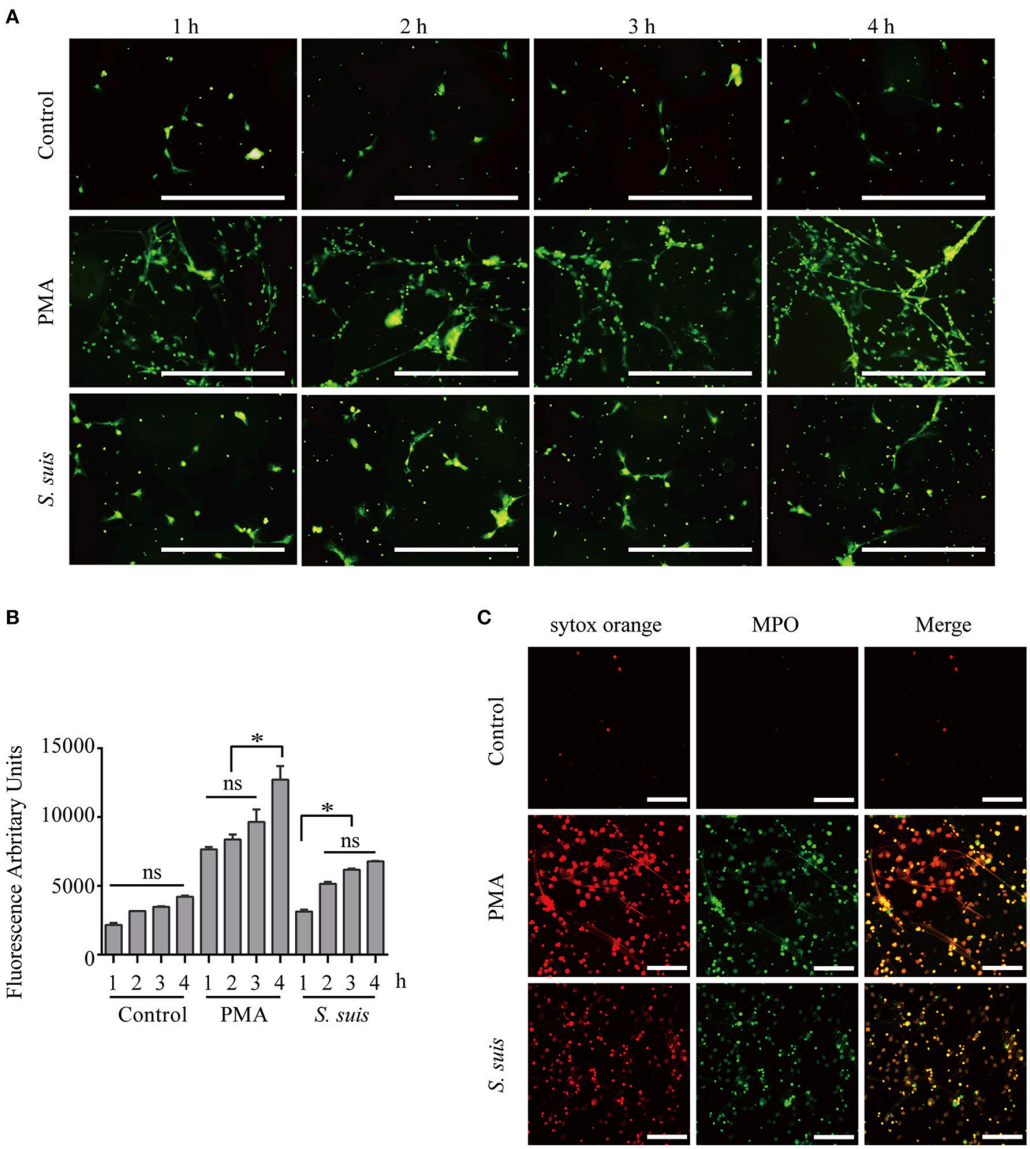
Induction of NET formation by PMA or *S. suis*. Purified murine neutrophils were incubated with an *S. suis* strain at a MOI = 2 or with PMA (100 nM) in the presence of the extracellular DNA dye SYTOX Green. At 1–4 h post-incubation, NET formation was visualized under a fluorescence microscope using the 20× objective (OLYMPUS IX70) **(A)** and the absorbance was measured with a BioTek synergy HT plate reader at an excitation wavelength of 485 nm and emission wavelength of 530 nm (n = 5) **(B)**. Cells were incubated with 100 nM PMA, *S. suis* (MOI = 2), or PBS for 4 h and then incubated with SYTOX Orange and a Ms mAb against myeloperoxidase (FITC). Finally, these cells were analyzed with LSM 880 confocal microscope and ZEN 2.3 LITE software **(C)**. The scale bars shown in the figure represent 100 μm. *Significant difference, ns, no significant difference. Data are presented as means, and error bars represent ± standard deviations.

Mouse bone marrow neutrophils were freshly isolated Figure S1, treated with 100 nM PMA or *S. suis* (MOI = 2) for different times and visualized by staining with the extracellular nucleic acid dye SYTOX Green to observe NET formation and compare the similarities and difference in NETs formation induced by PMA stimulation and *S. suis* infection Figures 1A,B. During NET formation, neutrophil elastase (NE) and myeloperoxidase (MPO) were released from the azurophilic granules, which are required for PMA-induced NET formation (6, 18). Additionally, NE and MPO co-localize with DNA during NETs formation (18). The structure was further stained with SYTOX Orange and FITC-conjugated MPO antibodies to confirm whether the extracellular staining for nucleic acid was derived from NETs,. The obvious co-localization of SYTOX Orange and MPO antibodies indicated that the extracellular nucleic acid dye was a marker of NET formation in this system Figure 1C.

Furthermore, fluorescence microscopy analysis and a quantitative analysis of the fluorescence intensity revealed noticeable NET induction 1 h after the incubation with PMA, and more NETs appeared after 3 h Figure 1. *S. suis* also induced NET formation at 1 h post-infection, but significantly fewer NETs formed than after stimulation with PMA Figure 1. This finding was different from our previous observation that *S. suis* induced very obvious NET formation at a MOI = 10 (5), indicating that significant NET induction required a high level of *S. suis* infection. In the present study, PMA stimulation induced very obvious NET formation at 4 h post-incubation, while *S. suis* infection at a MOI = 2 only induced low levels of NET formation. Therefore, PMA or *S. suis* induced large or small amounts of NET formation, respectively, at 4 h post-treatment using the indicated method and represent two different NET formation models for our analysis of NET formation Figure 1.

Total proteins were extracted from neutrophils treated with PMA, *S. suis*, or PBS for 4 h and analyzed by SDS-PAGE. Stimulation with PMA or *S. suis* produced similar changes compared to the mock-treated controls, but different changes were also observed in response to both treatments Figure S2. Therefore, we performed a label-free quantitative proteomic analysis of the differential changes in protein levels in neutrophils subjected to different stimulation protocols to produce to strong or weak NET formation.

### Label-free quantitative proteomic analysis of neutrophils stimulated with PMA

**Figure 2:**
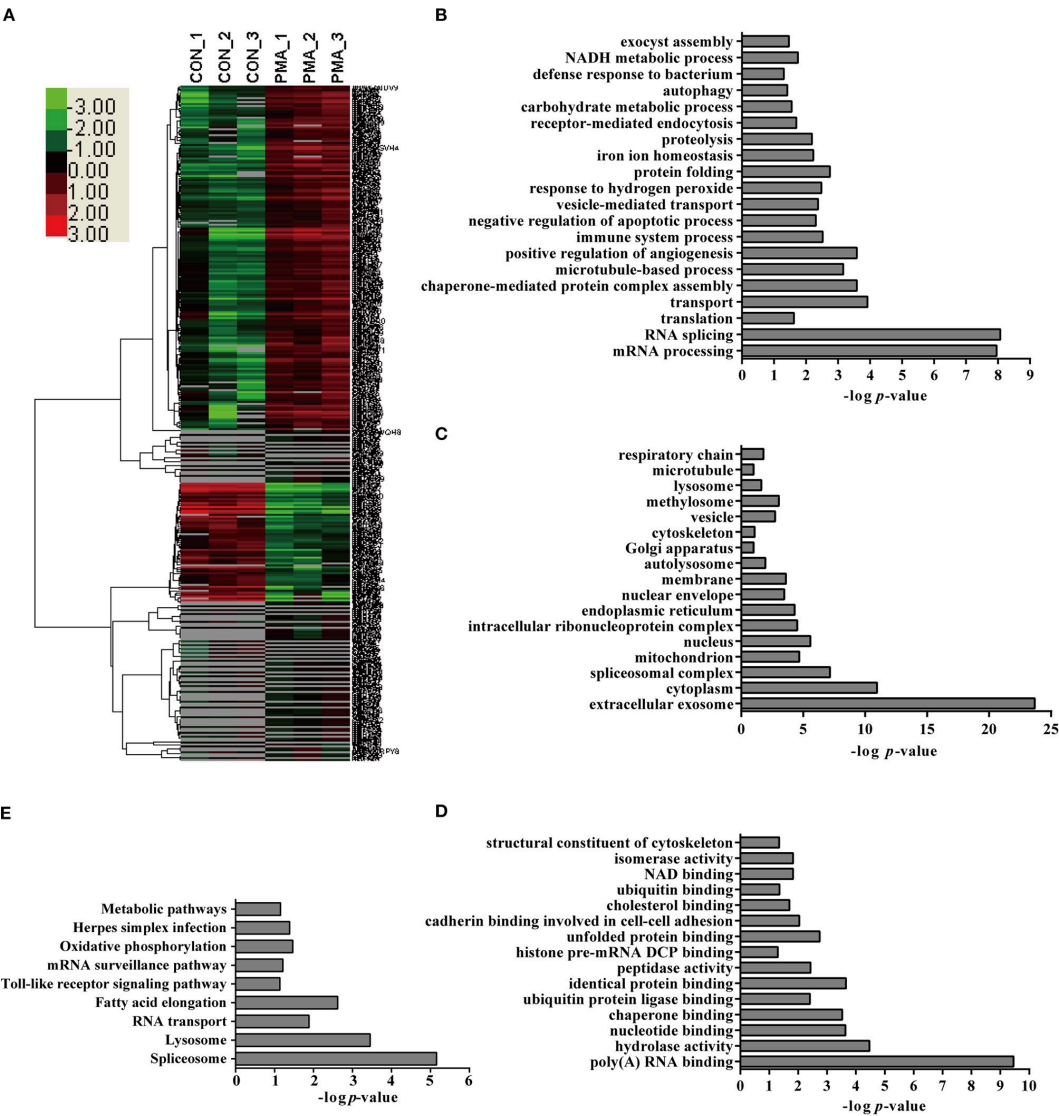
Bioinformatics analysis of DE proteins in NETs induced by PMA compared to the control. **(A)** A cluster analysis was performed based on the 347 DE proteins identified by a label-free quantitative proteomic analysis of neutrophils stimulated with PMA compared to the control. The color legend is shown on the left; the color scale ranges from saturated green for log ratios −3.0 and above to saturated red for log ratios 3.0 and above. Red indicates a higher expression level, and green indicates a lower expression level than in a normal sample. All of these DE proteins were subjected to an analysis with DAVID software at http://david.abcc.ncifcrf.gov for functional characterization. Biological processes **(B)**, cellular components **(C)**, molecular functions **(D)**, and KEGG pathways **(E)** of these DE proteins are shown.

PMA is a well-recognized NET inducer, so we first studied protein expression in neutrophils in response to PMA stimulation using a label-free quantitative proteomic analysis in the present study. Compared with control neutrophils, 1,558 unique peptides corresponding to 347 neutrophil proteins showed significant up- or down-regulation in response to PMA stimulation because the amounts of these proteins changed by more than 2-fold or < 0.5-fold at a *p* < 0.05 Datas S1–S3. These differentially expressed (DE) proteins included 97 down-regulated proteins and 250 up-regulated proteins in neutrophils stimulated with PMA for 4 h Figure 2A. Four proteins were randomly selected for a comparison of the expression by Western blot analysis to confirm the changes in protein expression during NET induction, and similar changes were observed to the label-free quantitative proteomic analysis Figure S3A. An analysis of the biological processes in which these DE proteins are involved indicated role in the mRNA metabolic process, RNA splicing, transport, translation, immune system process, negative regulation of apoptotic process, NADH metabolic process, proteolysis, etc. Figure 2B. An analysis of the cellular components indicated that these DE proteins were mainly distributed in the extracellular exosome, cytoplasm, spliceosomal complex, etc. Figure 2C. An analysis of molecular functions revealed that these proteins might participate in nucleotide binding, poly(A) RNA binding, hydrolase activity, peptidase activity, etc. Figure 2D. Further characterization with a KEGG pathway analysis indicated that the DE proteins were mainly involved in the spliceosome, lysosome, RNA transport, Toll-like receptor signaling pathway, oxidative phosphorylation, etc. Figure 2E.

Interestingly, several pathways involved in the NADPH metabolic process and apoptotic process, which were reported to be involved in NET formation (4, 19), were detected in this analysis, suggesting that the proteomics analysis provided information about NET formation. Additionally, the analysis also identified proteins involved in protein synthesis, immune system process, hydrolase activity, and peptidase activity. Undoubtedly, the contributions of these proteins to NET formation require further analysis.

### Label-free quantitative proteomic analysis of neutrophils infected with *S. suis*

**Figure 3:**
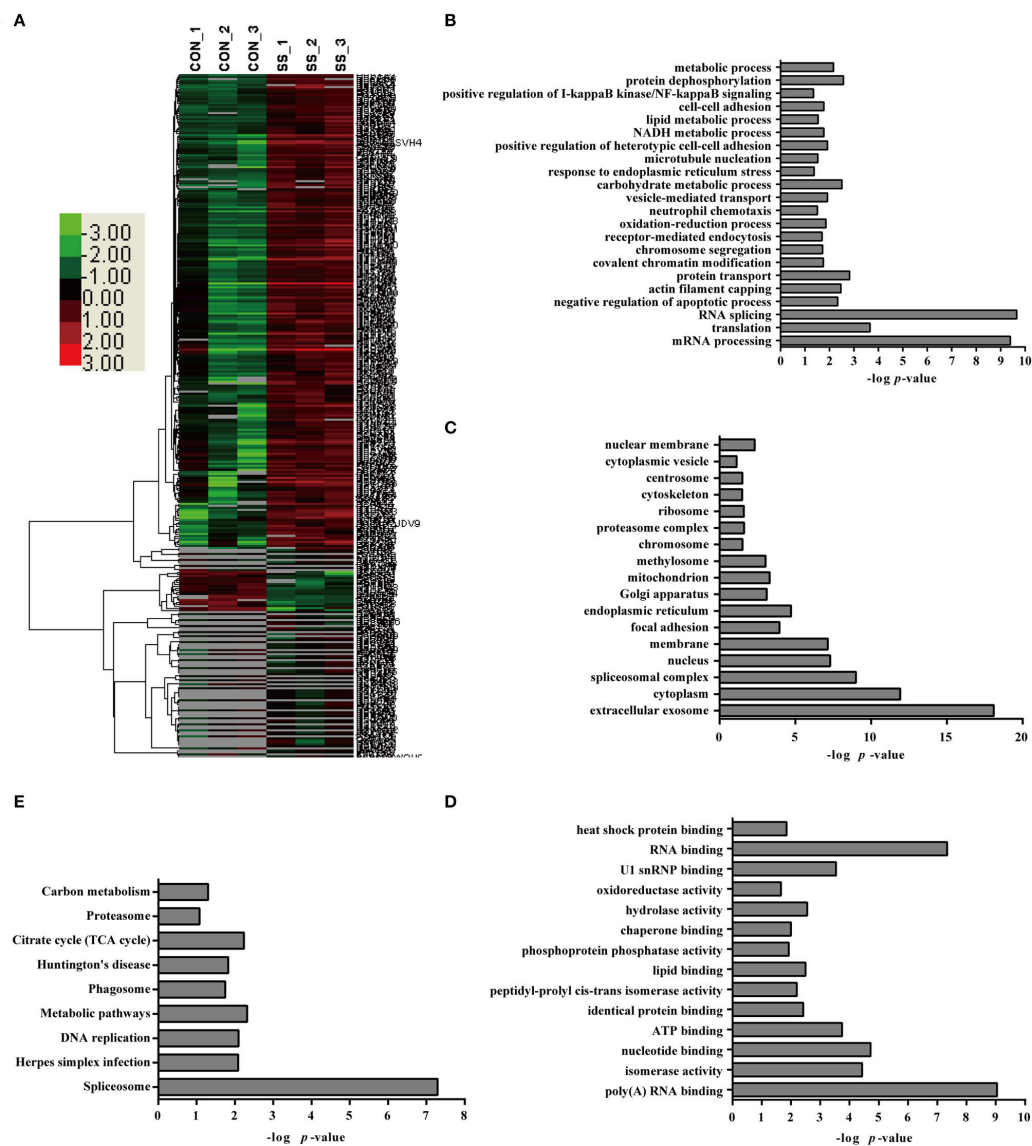
Bioinformatics analysis of DE proteins in NETs induced by *S. suis* infection compared to the control. **(A)** A cluster analysis of the 341 DE proteins identified by a label-free quantitative proteomic analysis of neutrophils infected with *S. suis* compared to the control was performed. The color legend is shown on the left; the color scale ranges from saturated green for log ratios −3.0 and above to saturated red for log ratios 3.0 and above. Red indicates a higher expression level, and green indicates a lower expression level than in a normal sample. All of these DE proteins were subjected to an analysis using DAVID software at http://david.abcc.ncifcrf.gov for functional characterization. Biological processes **(B)**, cellular components **(C)**, molecular functions **(D)**, and KEGG pathways **(E)** of these DE proteins are shown.

A comparative quantitative proteomic analysis of the proteins from the *S. suis*-infected and control groups yielded 1,607 unique peptides corresponding to 341 proteins that were identified as significantly up- or down-regulated because the amounts of these proteins showed more than a 2-fold or less than a 0.5-fold change with a *p* < 0.05 Datas S1–S3. Of these proteins, 46 were down-regulated and 295 were up-regulated in neutrophils infected with *S. suis* for 4 h Figure 3. Interestingly, the bioinformatics analysis indicated somewhat similar changes in the neutrophils in response to *S. suis* infection as to PMA stimulation, and included proteins involved NADPH metabolic process, actin polymerization, and apoptotic process Figure 3, which have been reported to be involved in NET formation (4, 19).

### Specific proteins are expressed in neutrophils in response to *S. suis* infection and PMA stimulation

**Figure 4:**
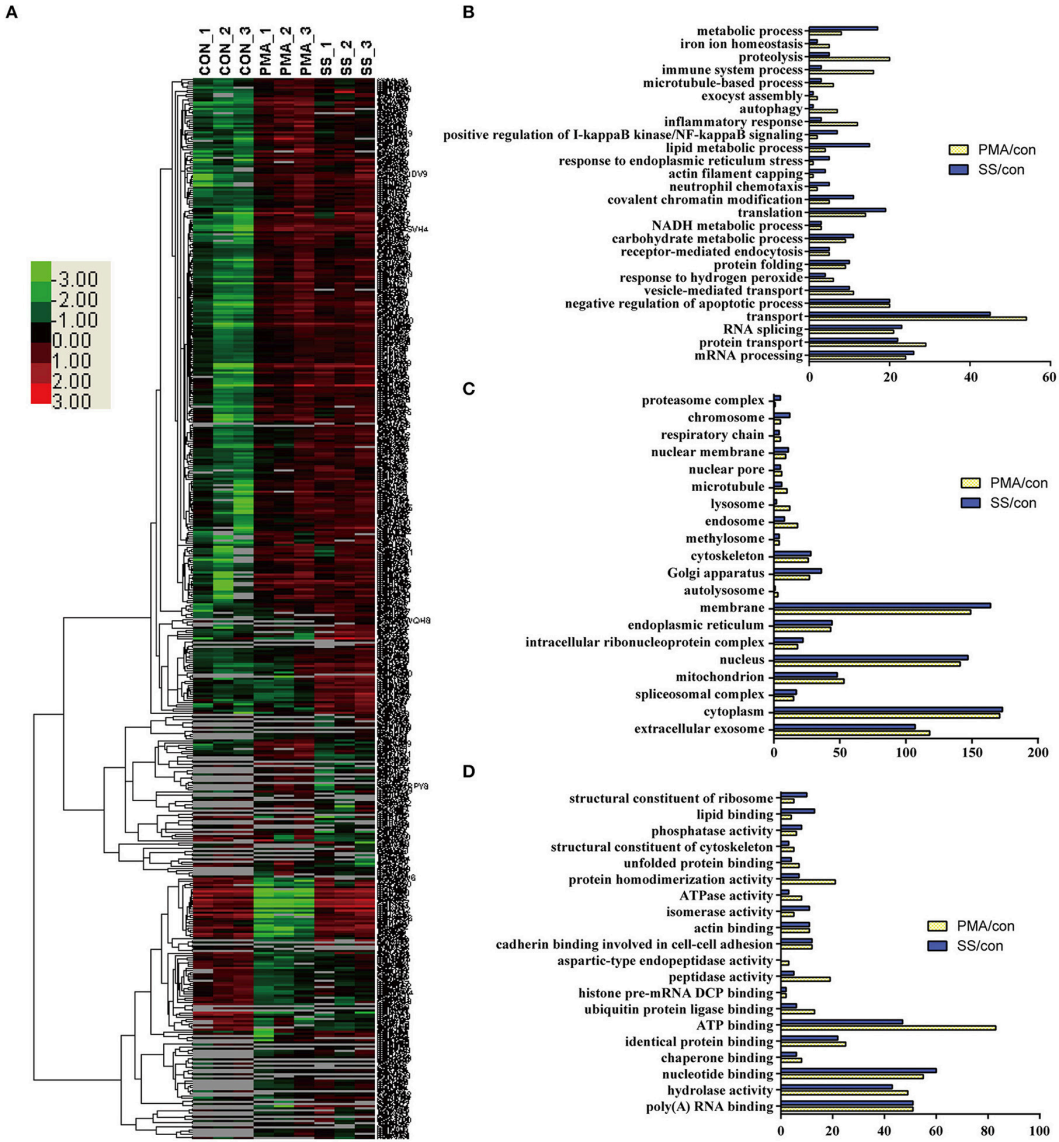
Comparison of DE proteins in NETs induced by PMA or *S. suis* infection compared to the control. **(A)** The DE proteins in NETs induced by PMA or *S. suis* infection were compared to the control and subjected to a cluster analysis. The color legend is shown on the left; the color scale ranges from saturated green for log ratios −3.0 and above to saturated red for log ratios 3.0 and above. Red indicates a higher expression level, and green indicates a lower expression level than in a normal sample. Biological processes **(B)**, cellular components **(C)**, and molecular functions **(D)** of these DE proteins and the number of proteins involved in the corresponding character are shown.

Because both *S. suis* infection and PMA stimulation induced NET formation in neutrophils, profiling of the DE proteins in cells stimulated with both treatments would facilitate the identification of the essential proteins involved in NET induction. An analysis of the biological processes in which the DE proteins were involved in cells infected with *S. suis* infection and stimulated with PMA compared with the control showed that a similar numbers of proteins were involved in the mRNA metabolic process, RNA splicing, protein transport, negative regulation of apoptotic process, receptor-mediated endocytosis, NADH metabolic process, etc. Figures 4A,B. However, the numbers of proteins involved in the metabolic process, proteolysis, immune system process, and lipid metabolic process were obviously different in both treated cell populations compared with the control Figure 4B. An analysis of the cellular components indicated that most of the DE proteins from cells stimulated with the two treatments displayed a similar distribution Figure 4C. An analysis of the molecular functions indicated that similar numbers of proteins were involved in nucleotide binding, RNA binding, hydrolase activity, and actin binding, and identical protein binding changes were observed in response to both *S. suis* infection and PMA stimulation for 4 h Figure 4D. Interestingly, among the DE proteins identified in neutrophils infected with *S. suis*, 52 % (176/341) were also DE proteins identified in neutrophils treated with PMA. Similarly, 51 % (176/347) of DE proteins identified in neutrophils treated with PMA were also detected in the *S. suis* infection group Figure 4A. These DE proteins showed similar changed in response to *S. suis* and PMA and thus might be required for NET formation. For example, the identified proteins involved in the negative regulation of apoptotic process suggested a potential role in NETosis, because apoptosis might function as a backup program of cell death for NETosis when NET formation is prevented (19).

Nineteen proteins with peptidase activity exhibited alterations in response to PMA stimulation; however, only 6 proteins (Sec11c, Blmh, Eif3f, Metap2, Psme3, and Thop1) were altered in response to an *S. suis* infection Figure 4D and Table S1. In comparison, Asprv1, Ctsc, Ctsd, Ctse, Ctsz, Ide, Ltf, Lonp1, Mcpt8, Mmp25, Mmp8, Mmp9, and Pitrm1 were only differentially expressed in the PMA-treated cells, but not in infected cells, compared with the control Table S1. This sharp contrast was also observed in the proteins involved in ubiquitin protein ligase binding; 13 proteins were differentially expressed in response to PMA stimulation, while only 6 were altered in response to an *S. suis* infection Figure 4D. Additional differences were observed by directly comparing protein expression during NET formation induced by PMA with that induced by *S. suis* infection Figure S4. Because PMA induced very obvious NET formation while *S. suis* (MOI = 2) induced only weak NET formation Figure 1, the associations between these peptidases and the level of NET formation are worth exploring in future studies.

### PKC, NADPH oxidase, and MPO are required for NET formation induced by PMA or *S. suis* infection, but actin polymerization is differentially required

**Figure 5:**
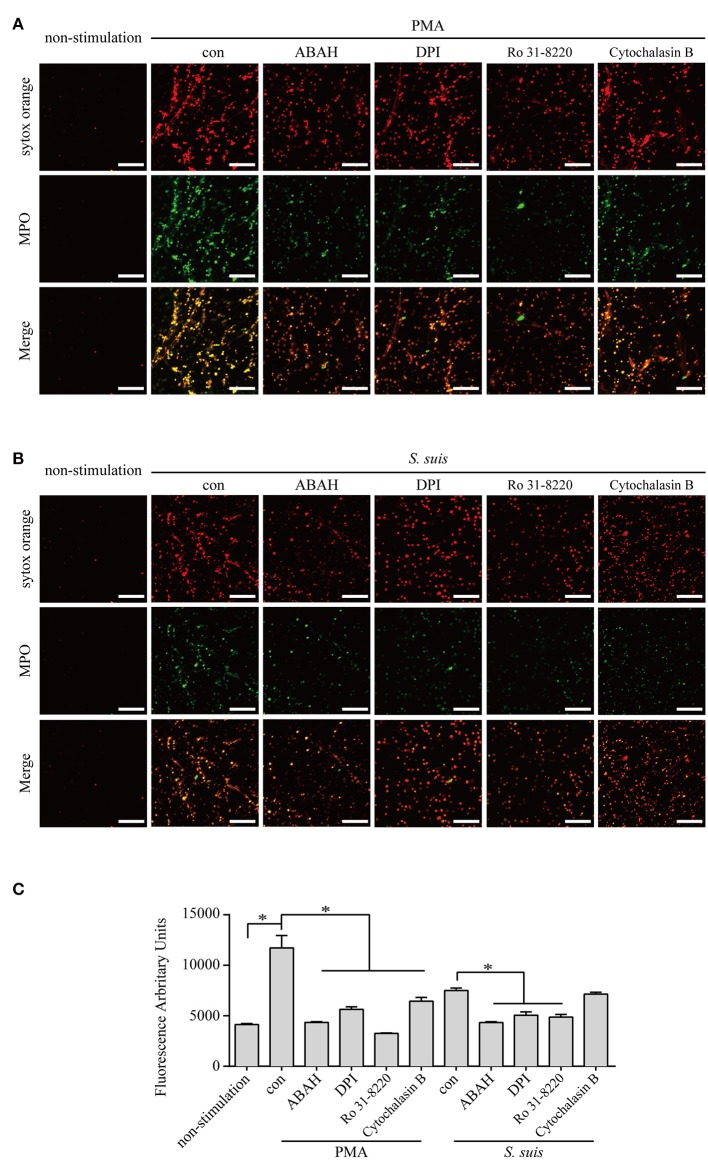
NET induction by PMA or *S. suis* in cells treated with various inhibitors. Purified murine neutrophils were incubated with PMA (100 nM) **(A)** or *S. suis* at MOI = 2 **(B)** in the presence of following inhibitors: 1 μM Ro 31-8220 (an inhibitor of PKC), 5 μM DPI (an inhibitor of ROS), 100 μM ABAH (an inhibitor of MPO), or 10 μM cytochalasin B (an actin polymerization inhibitor). NET formation was visualized by staining with the extracellular nucleic acid dye SYTOX Orange (100 nM) for 10 min and a subsequent incubation with a FITC-labeled Ms mAb against myeloperoxidase for 1 h. NET formation was visualized with an LSM 880 confocal microscope (ZEISS) and ZEN 2.3 LITE software (ZEISS). Cells were incubated with SYTOX Green for 10 min, and then the absorbance was measured at 485/530 nm using a BioTek synergy HT plate reader (*n* = 5) to further quantitatively evaluate the effects of these inhibitors on NET formation **(C)**. The scale bars shown in the figure represent 100 μm; *Significant difference, ns, no significant difference. Data are presented as means, and error bars indicate ± standard deviations.

*S. suis* induces NET formation *in vitro* and *in vivo* (5, 39, 41, 42). However, researchers have not clearly identified the signaling pathways required for NET formation induced by *S. suis*. Because more than half of DE proteins identified in neutrophils infected with *S. suis* showed similar changes to cells stimulated with PMA Figure 4, we considered the role of signaling in PMA-induced NETs on NET formation induced by *S. suis*. PMA-induced NET formation depends on PKC, NADPH oxidase and MPO (4). In the present study, Ro 31-8220 (an inhibitor of PKC), DPI (an inhibitor of ROS), or ABAH (an inhibitor of MPO) inhibited NET formation induced by PMA or *S. suis* Figure 5, indicating that *S. suis*-induced NET formation also required the activation of PKC, NADPH oxidase, and MPO. The finding was somewhat different from NET formation induced by Group B *Streptococcus*, which requires PKC and MPO activity, but only partially requires NADPH oxidase (17).

Actin polymerization is responsible for NE translocation to the nucleus (21), which is required for NET formation induced by several stimuli (21, 48), including PMA Figure 5. In contrast, an inhibitor of actin polymerization did not obviously inhibit NET formation induced by *S. suis* Figure 5, suggesting that the failure to regulate actin polymerization in neutrophils treated with *S. suis* at a low MOI is one of explanation for the weak formation of NETs.

Based on these findings, similar pathways were required for NET formation induced by PMA or *S. suis*, but some differences in NET formation induced by the two stimuli remain.

### MMP-8 activity is inversely correlated with NET formation induced by PMA or *S. suis* infection

Although NET formation induced by either PMA or *S. suis* infection depended on the PKC pathway Figure 5, a substantial difference in the amount of NETs formed was observed Figure 1. Interestingly, 14 proteins involved in peptidase activity were only differentially expressed in the cells treated with PMA but not in cells infected with *S. suis* compared with the control Figure 4D and Table S1. Among these peptidases, several matrix metalloproteinases exhibited noticeable changes in response to PMA, including MMP-8 (Figure S3B and Table S1. Therefore, we aimed to determine the association of NET formation with MMP-8 activity using an inhibitor.

**Figure 6:**
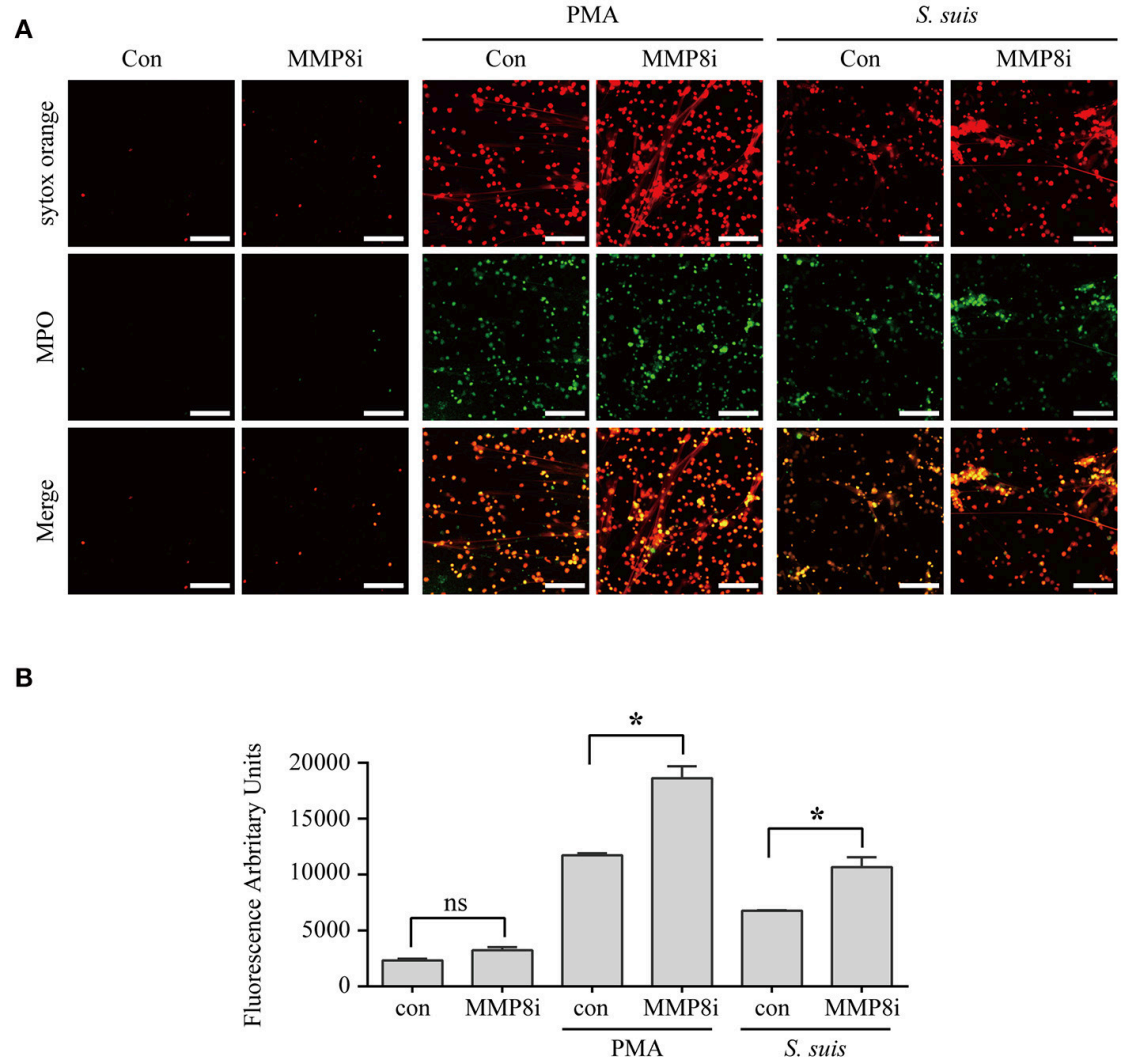
MMP-8 activity inhibited NET formation induced by PMA or *S. suis* infection. Purified murine neutrophils were incubated with *S. suis* (MOI = 2) or PMA (100 nM) in the presence of an MMP-8 inhibitor. NET formation was visualized by staining cells with the extracellular nucleic acid dye SYTOX Orange (100 nM) and a FITC-labeled Ms mAb against myeloperoxidase, followed by an analysis with an LSM 880 confocal microscope and ZEN 2.3 LITE software **(A)**. Cells were also incubated with SYTOX Orange for 10 min, and then the absorbance was measured at 485/530 nm using a BioTek synergy HT plate reader (*n* = 5) **(B)**. The scale bars shown in the figure represent 100 μm. *Significant difference, ns, no significant difference. Data are presented as means, and error bars indicate ± standard deviations.

The MMP-8 inhibitor significantly enhanced the NET formation induced by both PMA and *S. suis* Figure 6, indicating that MMP-8 activity was inversely correlated with NET formation induced by PMA or *S. suis* infection. Therefore, low levels of MMP-8 in PMA-treated neutrophils might account for the large number of NETs that formed, although further studies are required to elucidate the mechanism by which MMP-8 activity inhibits NET formation. In addition, the results also provided an explanation for the observation that an infection with *S. suis* did not induce robust NETs formation, although it activated PKC signaling to the same extent as PMA stimulation.

Currently, two types of NET formation have been described (49); one is often designated suicidal NETosis because it leads to the loss of plasma membrane integrity and neutrophil death (3), and the other is described as “vital NETosis” because the neutrophils remained intact and functional after ejecting their DNA (47, 50). Based on the findings from the present study, *S. suis*-induced NETs are likely defined as suicidal NETosis and differ from the vital NETosis induced by *Staphylococcus aureus* (50), which did not depend on ROS (47). Furthermore, the signaling pathway required for *S. suis*-induced NET formation was somewhat different from PMA or Group B *Streptococcus*-induced NET formation, indicating that the signaling pathways required for NET formation are complicated and may depend on the stimulus.

*Streptococcus suis* obviously induces NET formation at a high MOI (MOI = 10) (5), but the high level of a cytotoxin (suilysin) potentially causes the death of neutrophils (51), which might interfere with observations of NET formation. In addition, high concentrations of bacterial proteins would also interfere with the proteomic analysis of the host response to the infection. Therefore, we chose an infection at a low MOI (MOI = 2) to study the formation of NETs, which was significantly blocked by the inhibitors of PKC signaling Figure S4, indicating that infection with *S. suis* at a low MOI was a feasible method to study NET formation. When the MS/MS data were searched against the *S. suis* database, < 5% of total peptides were associated with bacterial proteins. Thus, an infection at an MOI = 2 significantly decreased the effects of bacterial proteins on the results of the proteomic analysis of host proteins.

In addition, the enolase of *Streptococcus pneumoniae* was reported to induce NET formation (52), and *S. suis* expresses enolase on the cell surface (53), but researchers have not yet determined whether it induces NETs formation. However, the high concentration of this protein on bacterial cells at a high MOI may also provide an explanation for why a high bacterial concentration induces more obvious NET formation. Thus, during the early stages of infection, a lower amount of *S. suis* is unable to induce the formation of a sufficient number of NETs to kill the bacteria, which might explain why *S. suis* is able to evade the host immune response during the early stages of infection.

## Data access

The LC-MS/MS data have been submitted to the integrated proteome resources iProX (http://www.iprox.orgunder Project ID number IPX0001094000).

## Author contributions

The experiments were performed mainly by XW and JZ, and some experiments were performed with the help of CC, XT, LF. LH and AZ performed the data analysis. The study was designed by LH and AZ.

### Conflict of interest statement

The authors declare that the research was conducted in the absence of any commercial or financial relationships that could be construed as a potential conflict of interest.

## Supplementary material

Figure S1 Analysis of the purity of the isolated neutrophils by flow cytometry. The isolated mouse bone marrow neutrophils were fixed with 4% paraformaldehyde, incubated with a rat anti-mouse CD16/32 antibody, and then stained with allophycocyanin (APC)-conjugated anti-mouse Ly-6G. Subsequently, the cells were analyzed using a BD FACSVerse™ flow cytometer and FlowJo 7.6.1 software.

Figure S2 SDS-PAGE analysis of total proteins extracted from NET structures induced by PMA or *S. suis*. The total proteins extracted from neutrophils that were mock-treated with PBS as a control were also subjected to the analysis.

Figure S3 Western blot analysis of selected DE proteins identified using the label-free quantitative proteomic analysis. GAPDH served as a reference control. **(A)** Western blot analysis of four selected DE proteins: PGRP-S, WDR5, lysozyme and calpain. **(B)** Western blot analysis of MMP-8, a DE protein identified using the label-free quantitative proteomic analysis.

Figure S4 Bioinformatics analysis of DE proteins in NETs induced by PMA compared to the *S. suis* infection. **(A)** A cluster analysis of the 97 DE proteins identified using a label-free quantitative proteomic analysis of neutrophils induced with PMA was performed and compared to the cells infected with *S. suis*. The color legend is shown on the left; the color scale ranges from saturated green for log ratios−3.0 and above to saturated red for log ratios 3.0 and above. Red indicates a higher expression level, and green indicates a lower expression level than in a normal sample. All of these DE proteins were subjected to an analysis using DAVID software at http://david.abcc.ncifcrf.gov for functional characterization. Biological processes **(B)**, cellular components **(C)**, molecular functions **(D)**, and KEGG pathways **(E)** of these DE proteins are shown.

Table S1 DE proteins involved in peptidase activity.

Data S1 Peptides identified using MS.

Data S2 Proteins identified using MS.

Data S3 The differential expression of proteins identified using the label-free quantitative proteomic analysis.

## References

[1] Nathan C. Neutrophils and immunity: challenges and opportunities. Nat Rev Immunol. (2006) 6:173–82. 10.1038/nri1785 16498448

[2] Scapini P Cassatella MA. Social networking of human neutrophils within the immune system. Blood (2014) 124:710–9. 10.1182/blood-2014-03-453217 24923297

[3] Brinkmann V Reichard U Goosmann C Fauler B Uhlemann Y Weiss DS. Neutrophil extracellular traps kill bacteria. Science (2004) 303:1532–5. 10.1126/science.1092385 15001782

[4] Fuchs TA Abed U Goosmann C Hurwitz R Schulze I Wahn V. Novel cell death program leads to neutrophil extracellular traps. J Cell Biol. (2007) 176:231–41. 10.1083/jcb.200606027 17210947 PMC2063942

[5] Zhao JQ Pan S Lin L Fu L Yang C Xu ZM. *Streptococcus suis* serotype 2 strains can induce the formation of neutrophil extracellular traps and evade trapping. Fems Microbiol Lett. (2015) 362:fnv022. 10.1093/femsle/fnv022 25673283

[6] Metzler KD Fuchs TA Nauseef WM Reumaux D Roesler J Schulze I. Myeloperoxidase is required for neutrophil extracellular trap formation: implications for innate immunity. Blood (2011) 117:953–9. 10.1182/blood-2010-06-290171 20974672 PMC3035083

[7] Saitoh T Komano J Saitoh Y Misawa T Takahama M Kozaki T. Neutrophil extracellular traps mediate a host defense response to human immunodeficiency virus-1. Cell Host Microbe (2012) 12:109–16. 10.1016/j.chom.2012.05.015 22817992

[8] Jenne CN Wong CH Zemp FJ Mcdonald B Rahman MM Forsyth PA. Neutrophils recruited to sites of infection protect from virus challenge by releasing neutrophil extracellular traps. Cell Host Microbe (2013) 13:169–80. 10.1016/j.chom.2013.01.005 23414757

[9] Jaillon S Peri G Delneste Y Fremaux I Doni A Moalli F. The humoral pattern recognition receptor PTX3 is stored in neutrophil granules and localizes in extracellular traps. J Exp Med. (2007) 204:793–804. 10.1084/jem.20061301 17389238 PMC2118544

[10] Clark SR Ma AC Tavener SA Mcdonald B Goodarzi Z Kelly MM. Platelet TLR4 activates neutrophil extracellular traps to ensnare bacteria in septic blood. Nat Med. (2007) 13:463–9. 10.1038/nm1565 17384648

[11] Mcdonald B Urrutia R Yipp BG Jenne CN Kubes P. Intravascular neutrophil extracellular traps capture bacteria from the bloodstream during sepsis. Cell Host Microbe (2012) 12:324–33. 10.1016/j.chom.2012.06.011 22980329

[12] Parker H Dragunow M Hampton MB Kettle AJ Winterbourn CC. Requirements for NADPH oxidase and myeloperoxidase in neutrophil extracellular trap formation differ depending on the stimulus. J Leukoc Biol. (2012) 92:841–9. 10.1189/jlb.1211601 22802447

[13] Gray RD Lucas CD Mackellar A Li F Hiersemenzel K Haslett C. Activation of conventional protein kinase C (PKC) is critical in the generation of human neutrophil extracellular traps. J Inflamm. (2013) 10:12. 10.1186/1476-9255-10-12 23514610 PMC3643828

[14] Hakkim A Fuchs TA Martinez NE Hess S Prinz H Zychlinsky A. Activation of the Raf-MEK-ERK pathway is required for neutrophil extracellular trap formation. Nat Chem Biol. (2011) 7:75–7. 10.1038/nchembio.496 21170021

[15] Keshari RS Verma A Barthwal MK Dikshit M. Reactive oxygen species-induced activation of ERK and p38 MAPK mediates PMA-induced NETs release from human neutrophils. J Cell Biochem. (2013) 114:532–40. 10.1002/jcb.24391 22961925

[16] Gupta AK Giaglis S Hasler P Hahn S. Efficient neutrophil extracellular trap induction requires mobilization of both intracellular and extracellular calcium pools and is modulated by cyclosporine A. PLoS ONE (2014) 9:e97088. 10.1371/journal.pone.0097088 24819773 PMC4018253

[17] Kenny EF Herzig A Kruger R Muth A Mondal S Thompson PR. Diverse stimuli engage different neutrophil extracellular trap pathways. Elife (2017) 6:e24437. 10.7554/eLife.24437 28574339 PMC5496738

[18] Papayannopoulos V Metzler KD Hakkim A Zychlinsky A. Neutrophil elastase and myeloperoxidase regulate the formation of neutrophil extracellular traps. J Cell Biol. (2010) 191:677–91. 10.1083/jcb.201006052 20974816 PMC3003309

[19] Remijsen Q Vanden Berghe T Wirawan E Asselbergh B Parthoens E De Rycke R. Neutrophil extracellular trap cell death requires both autophagy and superoxide generation. Cell Res. (2011) 21:290–304. 10.1038/cr.2010.150 21060338 PMC3193439

[20] Xu Z Cai J Gao J White GCIII Chen F Ma YQ. Interaction of kindlin-3 and beta2-integrins differentially regulates neutrophil recruitment and NET release in mice. Blood (2015) 126:373–7. 10.1182/blood-2015-03-636720 26056166

[21] Metzler KD Goosmann C Lubojemska A Zychlinsky A Papayannopoulos V. A myeloperoxidase-containing complex regulates neutrophil elastase release and actin dynamics during NETosis. Cell Rep. (2014) 8:883–96. 10.1016/j.celrep.2014.06.044 25066128 PMC4471680

[22] Rodriguez-Espinosa O Rojas-Espinosa O Moreno-Altamirano MM Lopez-Villegas EO Sanchez-Garcia FJ. Metabolic requirements for neutrophil extracellular traps formation. Immunology (2015) 145:213–24. 10.1111/imm.12437 25545227 PMC4427386

[23] Kawakami T He J Morita H Yokoyama K Kaji H Tanaka C. Rab27a is essential for the formation of neutrophil extracellular traps (NETs) in neutrophil-like differentiated HL60 cells. PLoS ONE (2014) 9:e84704. 10.1371/journal.pone.0084704 24404184 PMC3880328

[24] Sollberger G Amulic B Zychlinsky A. Neutrophil extracellular trap formation is independent of *de novo* gene expression. PLoS ONE (2016) 11:e0157454. 10.1371/journal.pone.0157454 27310721 PMC4911059

[25] Segura M. *Streptococcus suis*: an emerging human threat. J Infect Dis. (2009) 199:4–6. 10.1086/594371 19016626

[26] Wertheim HF Nghia HD Taylor W Schultsz C. *Streptococcus suis*: an emerging human pathogen. Clin Infect Dis. (2009) 48:617–25. 10.1086/596763 19191650

[27] Gottschalk M Streptococcocis. In: Zimmerman J. Karriker L. Ramirez A. Schwartz K. Stevenson G. editors. Diseases of Swine, 10th ed. Ames, IA: Blackwell Publishing (2012). p. 841–55.

[28] Huong VT Ha N Huy NT Horby P Nghia HD Thiem VD. Epidemiology, clinical manifestations, and outcomes of *Streptococcus suis* infection in humans. Emerg Infect Dis. (2014) 20:1105–14. 10.3201/eid2007.131594 24959701 PMC4073838

[29] Goyette-Desjardins G Auger JP Xu J Segura M Gottschalk M. *Streptococcus suis*, an important pig pathogen and emerging zoonotic agent-an update on the worldwide distribution based on serotyping and sequence typing. Emerg Microbes Infect. (2014) 3:e45. 10.1038/emi.2014.45 26038745 PMC4078792

[30] Suankratay C Intalapaporn P Nunthapisud P Arunyingmongkol K Wilde H. *Streptococcus suis* meningitis in Thailand. Southeast Asian J Trop Med Public Health (2004) 35:868–76. 15916083

[31] Mai NT Hoa NT Nga TV Linh Le D Chau TT Sinh DX. *Streptococcus suis* meningitis in adults in Vietnam. Clin Infect Dis. (2008) 46:659–67. 10.1086/527385 19413493

[32] Huang YT Teng LJ Ho SW Hsueh PR. *Streptococcus suis* infection. J Microbiol Immunol Infect. (2005) 38:306–13. 16211137

[33] Smith TC Capuano AW Boese B Myers KP Gray GC. Exposure to *Streptococcus suis* among US swine workers. Emerg Infect Dis. (2008) 14:1925–7. 10.3201/eid1412.080162 19046523 PMC2634616

[34] Fowler HN Brown P Rovira A Shade B Klammer K Smith K. *Streptococcus suis* meningitis in swine worker, Minnesota, U*S*A. Emerg Infect Dis. (2013) 19:330–1. 10.3201/eid1902.120918 23460993 PMC3559051

[35] Tang J Wang C Feng Y Yang W Song H Chen Z. Streptococcal toxic shock syndrome caused by *Streptococcus suis* serotype 2. PLoS Med. (2006) 3:e151. 10.1371/journal.pmed.0030151 16584289 PMC1434494

[36] Yu H Jing H Chen Z Zheng H Zhu X Wang H. Human *Streptococcus suis* outbreak, Sichuan, China. Emerg Infect Dis. (2006) 12:914–20. 10.3201/eid1206.051194 16707046 PMC3373052

[37] Manzin A Palmieri C Serra C Saddi B Princivalli MS Loi G. *Streptococcus suis* meningitis without history of animal contact, Italy. Emerg Infect Dis. (2008) 14:1946–8. 10.3201/eid1412.080679 19046529 PMC2634631

[38] Callejo R Prieto M Salamone F Auger JP Goyette-Desjardins G Gottschalk M A typical *Streptococcus suis* in man, Argentina, 2013. Emerg Infect Dis. (2014) 20:500–2. 10.3201/eid2003.131148 24565286 PMC3944841

[39] De Buhr N Stehr M Neumann A Naim HY Valentin-Weigand P Von Kockritz-Blickwede M Identification of a novel DNase of *Streptococcus suis* (EndAsuis) important for neutrophil extracellular trap (NET) degradation during exponential growth. Microbiology (2015) 161(Pt 4):838–50. 10.1099/mic.0.000040 25667008

[40] Ma F Yi L Yu N Wang G Ma Z Lin H. *Streptococcus suis* serotype 2 biofilms inhibit the formation of neutrophil extracellular traps. Front Cell Infect Microbiol. (2017) 7:86. 10.3389/fcimb.2017.00086 28373968 PMC5357632

[41] Zhao J Lin L Fu L Han L Zhang A Neutrophils extracellular Taps play an important role in clearance of *Streptococcus suis in vivo*. Microbiol Immunol. (2016) 60:228–33. 10.1111/1348-0421.12367 26876770

[42] De Buhr N Reuner F Neumann A Stump-Guthier C Tenenbaum T Schroten H. Neutrophil extracellular trap formation in the *Streptococcus suis*-infected cerebrospinal fluid compartment. Cell Microbiol. (2017) 19:e12649. 10.1111/cmi.12649 27450700

[43] Wiener MC Sachs JR Deyanova EG Yates NA. Differential mass spectrometry: a label-free LC-MS method for finding significant differences in complex peptide and protein mixtures. Anal Chem. (2004) 76:6085–96. 10.1021/ac0493875 15481957

[44] Zhang A Chen B Yuan Z Li R Liu C Zhou H. HP0197 contributes to CPS synthesis and the virulence of *Streptococcus suis* via CcpA. PLoS ONE (2012) 7:e50987. 10.1371/journal.pone.0050987 23226442 PMC3511442

[45] Su X Johansen M Looney MR Brown EJ Matthay MA. CD47 deficiency protects mice from lipopolysaccharide-induced acute lung injury and Escherichia coli pneumonia. J Immunol. (2008) 180:6947–53. 10.4049/jimmunol.180.10.6947 18453616 PMC2771449

[46] Behnen M Moller S Brozek A Klinger M Laskay T. Extracellular acidification inhibits the ROS-dependent formation of neutrophil extracellular traps. Front Immunol. (2017) 8:184. 10.3389/fimmu.2017.00184 28293240 PMC5329032

[47] Bjornsdottir H Dahlstrand Rudin A Klose FP Elmwall J Welin A Stylianou M. Phenol-soluble modulin alpha peptide toxins from aggressive *Staphylococcus aureus* induce rapid formation of neutrophil extracellular traps through a reactive oxygen species-independent pathway. Front Immunol (2017) 8:257. 10.3389/fimmu.2017.00257 28337204 PMC5343011

[48] Neeli I Dwivedi N Khan S Radic M. Regulation of extracellular chromatin release from neutrophils. J Innate Immun. (2009) 1:194–201. 10.1159/000206974 20375577 PMC6951038

[49] Jorch SK Kubes P. An emerging role for neutrophil extracellular traps in noninfectious disease. Nat Med. (2017) 23:279–87. 10.1038/nm.4294 28267716

[50] Pilsczek FH Salina D Poon KK Fahey C Yipp BG Sibley CD. A novel mechanism of rapid nuclear neutrophil extracellular trap formation in response to *Staphylococcus aureus*. J Immunol. (2010) 185:7413–25. 10.4049/jimmunol.1000675 21098229

[51] Lecours MP Gottschalk M Houde M Lemire P Fittipaldi N Segura M. Critical role for *Streptococcus suis* cell wall modifications and suilysin in resistance to complement-dependent killing by dendritic cells. J Infect Dis. (2011) 204:919–29. 10.1093/infdis/jir415 21849289

[52] Mori Y Yamaguchi M Terao Y Hamada S Ooshima T Kawabata S. α-enolase of *Streptococcus pneumoniae* induces formation of neutrophil extracellular traps. J Biol Chem. (2012) 287:10472–81. 10.1074/jbc.M111.280321 22262863 PMC3323051

[53] Zhang A Xie C Chen H Jin M. Identification of immunogenic cell wall-associated proteins of *Streptococcus suis* serotype 2. Proteomics (2008) 8:3506–15. 10.1002/pmic.200800007 18686301

