# Supplementary figures and images for: A Label-Free Quantitative Proteomic Analysis of Mouse Neutrophil Extracellular Trap Formation Induced by Streptococcus suis or Phorbol Myristate Acetate (PMA)
Source: Front Immunol. 2018 Nov 13;9:2615. doi: 10.3389/fimmu.2018.02615 (PMC6282035; doi:10.3389/fimmu.2018.02615)

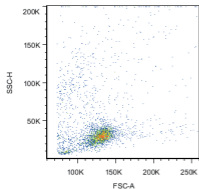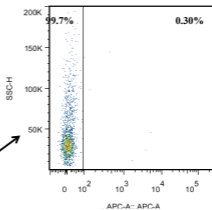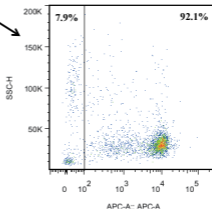

Supplement: Figure S1 — Analysis of the purity of the isolated neutrophils by flow cytometry. The isolated mouse bone marrow neutrophils were fixed with 4% paraformaldehyde, incubated with a rat anti-mouse CD16/32 antibody, and then stained with allophycocyanin (APC)-conjugated anti-mouse Ly-6G. Subsequently, the cells were analyzed using a BD FACSVerse™ flow cytometer and FlowJo 7.6.1 software. [file Image_1.PDF]

**A**

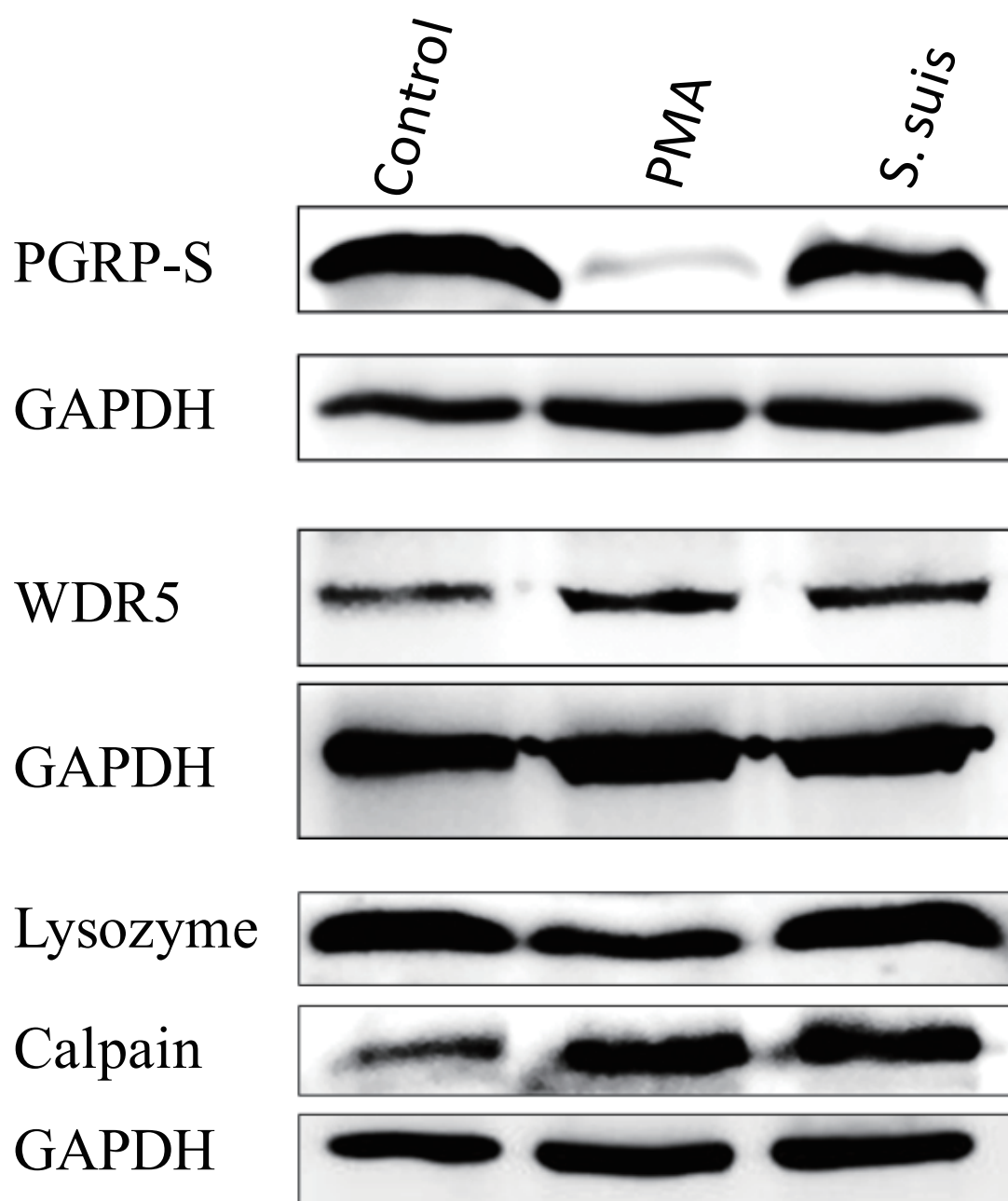

**B**

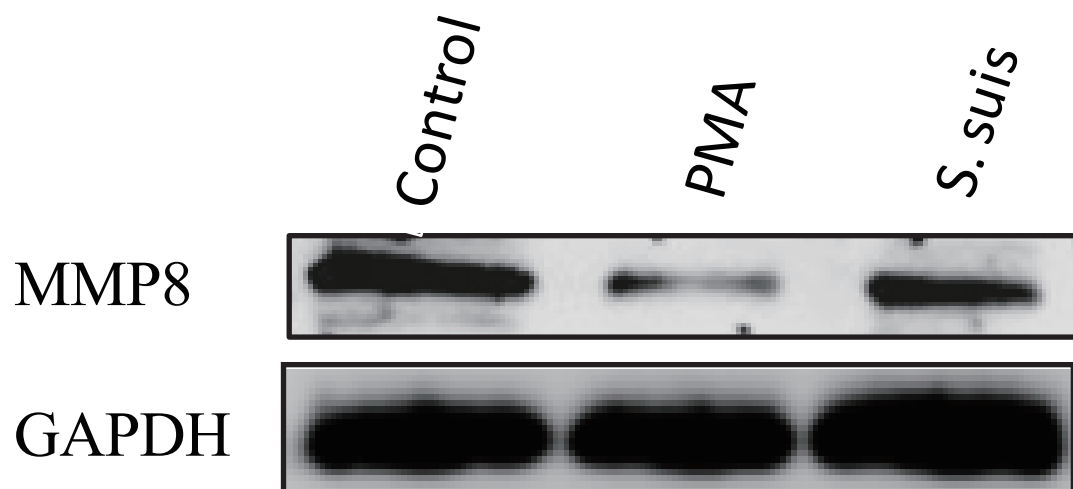

Supplement: Figure S3 — Western blot analysis of selected DE proteins identified using the label-free quantitative proteomic analysis. GAPDH served as a reference control. (A) Western blot analysis of four selected DE proteins: PGRP-S, WDR5, lysozyme and calpain. (B) Western blot analysis of MMP-8, a DE protein identified using the label-free quantitative proteomic analysis. [file Image_3.PDF]

A

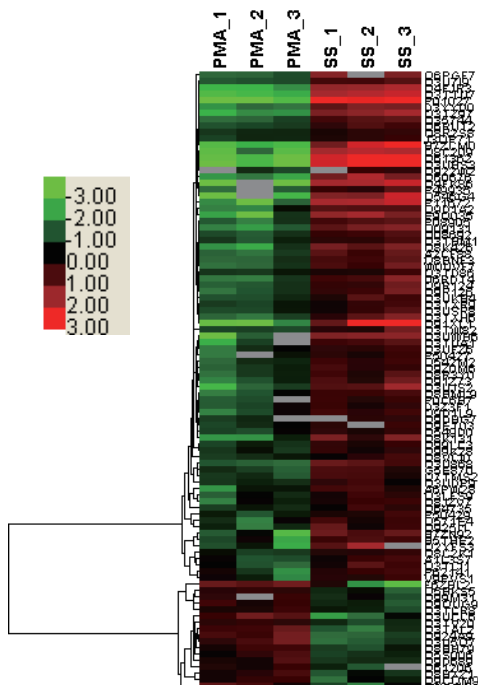

B

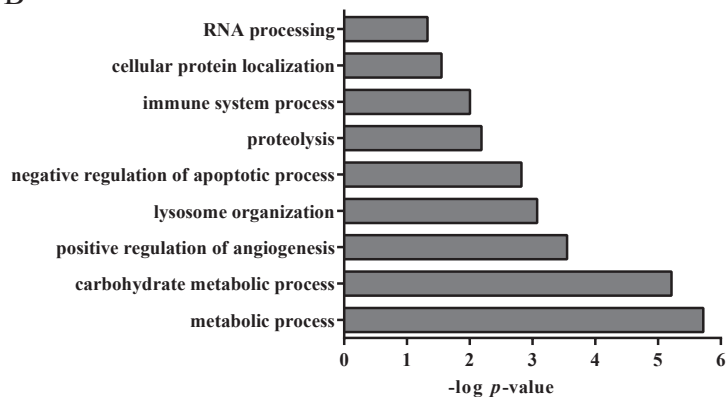

C

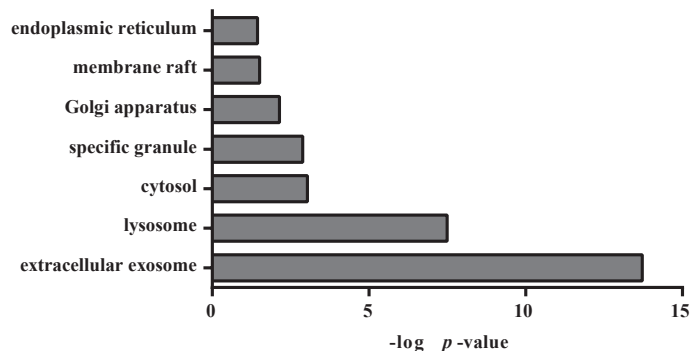

E

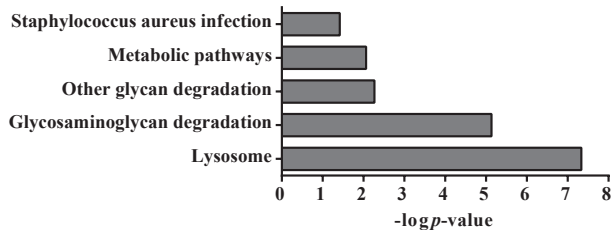

D

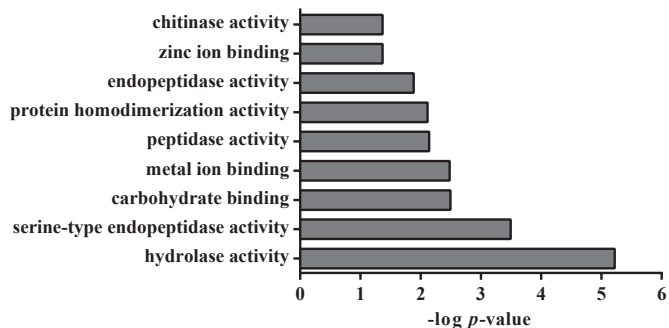

Supplement: Figure S4 — Bioinformatics analysis of DE proteins in NETs induced by PMA compared to the S. suis infection. (A) A cluster analysis of the 97 DE proteins identified using a label-free quantitative proteomic analysis of neutrophils induced with PMA was performed and compared to the cells infected with S. suis. The color legend is shown on the left; the color scale ranges from saturated green for log ratios−3.0 and above to saturated red for log ratios 3.0 and above. Red indicates a higher expression level, and green indicates a lower expression level than in a normal sample. All of these DE proteins were subjected to an analysis using DAVID software at http://david.abcc.ncifcrf.gov for functional characterization. Biological processes (B), cellular components (C), molecular functions (D), and KEGG pathways (E) of these DE proteins are shown. [file Image_4.PDF]
