# Supplementary material for: A Label-Free Quantitative Proteomic Analysis of Mouse Neutrophil Extracellular Trap Formation Induced by Streptococcus suis or Phorbol Myristate Acetate (PMA)
Source: Front Immunol. 2018 Nov 13;9:2615. doi: 10.3389/fimmu.2018.02615 (PMC6282035; doi:10.3389/fimmu.2018.02615)
Supplement: Figure S2 — SDS-PAGE analysis of total proteins extracted from NET structures induced by PMA or S. suis. The total proteins extracted from neutrophils that were mock-treated with PBS as a control were also subjected to the analysis. [file Image_2.PDF]

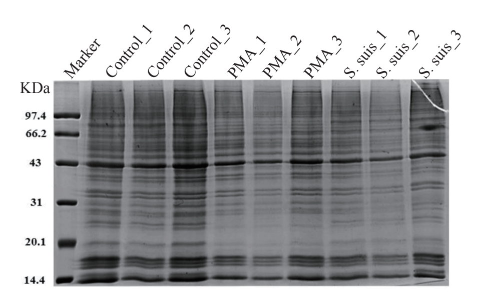

Marker
Control_1
Control_2
Control_3
PMA_1
PMA_2
PMA_3
S. suis_1
S. suis_2
S. suis_3
KDa
97.4
66.2
43
31
20.1
14.4
