# Supplementary material for: A Label-Free Quantitative Proteomic Analysis of Mouse Neutrophil Extracellular Trap Formation Induced by Streptococcus suis or Phorbol Myristate Acetate (PMA)
Source: Front Immunol. 2018 Nov 13;9:2615. doi: 10.3389/fimmu.2018.02615 (PMC6282035; doi:10.3389/fimmu.2018.02615)
Supplement: Table S1 — DE proteins involved in peptidase activity. [file Table_1.DOC]

**Table S1. DE proteins involved in peptidase activity**

| Protein | PMA/Con | | *S.suis*/Con | | PMA/*S.suis* | |
| --- | --- | --- | --- | --- | --- | --- |
|  | Fold change | p-value | Fold change | p-value | Fold change | p-value |
| SEC11 homolog C, signal peptidase complex subunit(Sec11c) | ∞ | 0.0016 | ∞ | 0.0344 |  |  |
| aspartic peptidase, retroviral-like 1(Asprv1) | 2.92 | 0.0103 |  |  |  |  |
| bleomycin hydrolase(Blmh) | 2.07 | 0.0093 | 2.42 | 0.0085 |  |  |
| cathepsin C(Ctsc) | ∞ | 0 |  |  |  |  |
| cathepsin D(Ctsd) | 0.30 | 0.0262 |  |  | 0.26 | 0.0002 |
| cathepsin E(Ctse) | 6.12 | 0.0057 |  |  |  |  |
| cathepsin Z(Ctsz) | 0.33 | 0.0404 |  |  |  |  |
| eukaryotic translation initiation factor 3, subunit F(Eif3f) | 2.40 | 0.0172 | 2.81 | 0.0027 |  |  |
| insulin degrading enzyme(Ide) | 4.85 | 0.0125 |  |  |  |  |
| lactotransferrin(Ltf) | 0.09 | 0.0019 |  |  | 0.06 | 0.0001 |
| lon peptidase 1, mitochondrial(Lonp1) | 2.57 | 0.0090 |  |  |  |  |
| mast cell protease 8(Mcpt8) | 0.04 | 0.0065 |  |  | 0.05 | 0.0001 |
| matrix metallopeptidase 25(Mmp25) | 0 | 0.0012 |  |  | 0 | 0.0001 |
| matrix metallopeptidase 8(Mmp8) | 0.05 | 0.0089 |  |  | 0.05 | 0.0001 |
| matrix metallopeptidase 9(Mmp9) | 0.06 | 0.0007 |  |  | 0.04 | 0.0003 |
| methionine aminopeptidase 2(Metap2) | 5.64 | 0.0099 | 6.11 | 0.0167 |  |  |
| pitrilysin metallepetidase 1(Pitrm1) | 0 | 0.0070 |  |  |  |  |
| proteaseome (prosome, macropain) activator subunit 3 (PA28 gamma, Ki)(Psme3) | 2.04 | 0.0326 | 2.51 | 0.0110 |  |  |
| thimet oligopeptidase 1(Thop1) | ∞ | 0.0039 | ∞ | 0.0016 |  |  |

“∞” indicated that the corresponding protein was not detected in the control group, but it was detected in the tested group.
